# Machine learning for predicting acute exacerbation and mortality in idiopathic inflammatory myopathy-associated interstitial lung disease

**DOI:** 10.3389/fmed.2026.1819663

**Published:** 2026-06-10

**Authors:** Saisai Lu, Mo Zheng, Shouliang Miao, Xiaofang Zhu, Guanxia Zhu, Lusi Ye, Dan Chen

**Affiliations:** 1 Department of Rheumatology, The First Affiliated Hospital of Wenzhou Medical University, Wenzhou, China; 2 Department of Radiology, The First Affiliated Hospital of Wenzhou Medical University, Wenzhou, China

**Keywords:** clinical indicators, idiopathic inflammatory myopathy, nomogram, poor prognosis, radiomics

## Abstract

**Objectives:**

Idiopathic inflammatory myopathy-associated interstitial lung disease (IIM-ILD) is a heterogeneous condition with high mortality. This study aimed to develop a nomogram integrating high-resolution computed tomography (HRCT) radiomics with clinical indicators to predict poor prognosis (AE-ILD or mortality) in IIM-ILD.

**Methods:**

167 patients were randomized into training (*n* = 118) and validation (*n* = 49) cohorts. In the training cohort, mRMR and LASSO algorithms identified optimal radiomic features. A combined nomogram was constructed using multivariate logistic regression. Performance was evaluated via ROC analysis, calibration curves, and decision curve analysis (DCA).

**Results:**

Independent predictors included rash (*p* = 0.007), absolute neutrophil count (*p* = 0.01), dyspnea (*p* = 0.03), and Alveolar-arterial oxygen gradient (*p* = 0.01). Twenty radiomic features were selected to calculate the Rad-score. The combined nomogram outperformed the clinical model in both cohorts. The training set achieved an AUC of 0.91 (sensitivity 0.90, specificity 0.84, and accuracy 0.86). The validation set showed an AUC of 0.88 (sensitivity 0.69, specificity 0.90, accuracy 0.82). Calibration curves (*p* > 0.05) and DCA confirmed the model’s reliability and clinical utility.

**Conclusion:**

The combined clinical-radiomics model effectively predicts poor prognosis in IIM-ILD, facilitating early identification and personalized intervention.

## 1 Introduction

Idiopathic inflammatory myopathy (IIM) is a group of autoimmune diseases characterized by proximal muscle weakness and chronic systemic inflammation, mainly including dermatomyositis, polymyositis, etc. (1). Pulmonary involvement is one of the important complications of IIM, among which interstitial lung disease (ILD) is the most common manifestation (2). IIM-ILD presents with diverse clinical outcomes, ranging from stable conditions to rapidly deteriorating “acute exacerbations” or even death (3).

In the course of IIM-ILD, acute exacerbation of ILD (AE-ILD) represents a sudden and severe clinical event characterized by rapid deterioration of respiratory function within a short period, often without identifiable triggers such as heart failure or pulmonary embolism (4, 5). AE-ILD is associated with high mortality rates and constitutes a significant cause of short-term mortality in patients (3, 6). Early identification of high-risk patients is crucial for initiating timely and aggressive treatment. However, the significant heterogeneity of IIM-ILD poses a major challenge to accurate prediction (7). Various laboratory indicators or clinical manifestations have been identified as risk factors for AE-ILD and mortality, such as ferritin, the level of KL6, and proximal muscle weakness (8–10). It has also suggested that the imaging manifestations of IIM patients may indicate the prognosis of ILD (11, 12).

High-resolution computed tomography (HRCT) can assess the extent and severity of the disease in IIM-ILD, and evaluate the therapeutic effect and prognosis of the disease (13, 14). However, the visual assessment of HRCT by clinicians is subjective and related to clinical experience. Therefore, there is concern that the diagnosis and treatment of ILD may be affected by the high variability among observers and within individual observers (15, 16). Radiomics is an emerging computational field that analyzes medical images by extracting vast amounts of quantitative data from them (17). In recent years, the development of radiomics and machine learning (ML) technologies has provided more possibilities for the objective assessment of IIM (18). By establishing correlations between imaging phenotypes and clinical outcomes, radiomics enables enhanced risk stratification. Nonetheless, the application of radiomics—particularly in combination with clinical factors—for predicting AE-ILD in IIM patients remains underexplored, representing a significant gap in current research.

This study aimed to develop and validate a combined model that integrates HRCT features with clinical indicators to predict the risk of AE-ILD in patients with IIM. We hypothesized that such a combined model would outperform models based solely on clinical or radiomic features, offering a robust tool for personalized risk assessment and early clinical intervention.

## 2 Materials and methods

### 2.1 Study design population

We retrospectively analyzed patients diagnosed with IIM-ILD at the First Affiliated Hospital of Wenzhou Medical University between January 1, 2019, and January 30, 2025. The diagnosis of IIM was established by rheumatologists according to the 2017 European League Against Rheumatism/American College of Rheumatology classification criteria (19) and the 239th European Neuromuscular Center International Workshop statement (20). The exclusion criteria were as follows: (1) age<18 years; (2) diagnosis of any malignant tumor; (3) coexistence of another defined autoimmune disease; or (4) lack of a definitive diagnosis regarding the site and pathogen of an active infection. In this study, the primary endpoint was defined as ‘poor prognosis’, which specifically refers to the occurrence of either acute exacerbation of ILD (AE-ILD) or mortality within the 6-month follow-up period.

**Figure 1:**
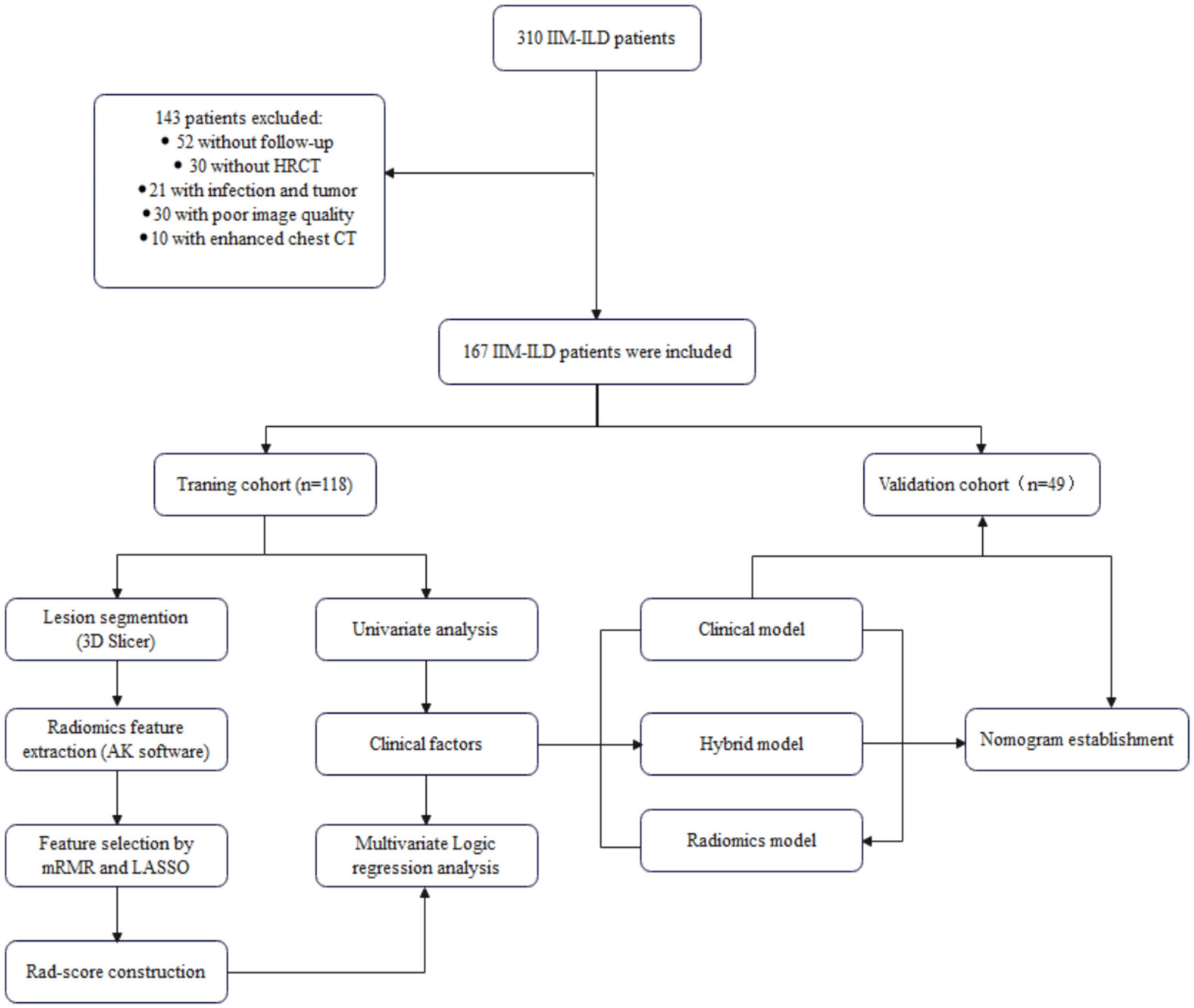
Flow chart of the study. IIM-ILD, Idiopathic inflammatory myopathy-associated lung disease. HRCT, High-resolution computed tomography. LASSO, Least absolute shrinkage and selection operator; mRMR, Maximum relevance–minimum redundancy.

During the first week of each patient’s hospitalization, baseline records were retrospectively collected, including demographic data, laboratory indicators, and radiological features. AE-ILD is diagnosed as acute deterioration or progresses to breathing difficulties, which typically lasts for 1 month; chest computed tomography shows new bilateral ground-glass opacities and/or consolidation superimposed on a background pattern consistent with ILD; and the deterioration cannot be fully explained by heart failure or fluid overload (5). Cutaneous manifestations were recorded as “rash” only when they met the typical IIM-associated patterns (Gottron’s sign, heliotrope rash, V-sign, or shawl sign) as documented by a board-certified rheumatologist, excluding non-specific or drug-induced rashes. The result data were collected from the medical records and obtained through outpatient follow-up or telephone communication. All patients were followed up for at least 6 months after diagnosis, or until their death. Follow-up period for this study concluded on July 30, 2025, oral consent was obtained by telephone. This study was conducted in accordance with the principles of the Declaration of Helsinki and was approved by the Institutional Review Board of Wenzhou Medical University Medical Ethics Committee (KY2024-R150). The research flowchart is shown in Figure 1.

In accordance with standard institutional clinical protocols, all available HRCT scans from baseline to the final follow-up were independently reviewed by two specialized thoracic radiologists. Any discrepancies were resolved through consensus with a third senior radiologist.

### 2.2 Data preprocessing and imputation

To ensure data quality, the following preprocessing procedures were implemented:

Outlier Handling: All laboratory values and quantitative clinical features underwent rigorous screening to identify and address potential outliers.

Missing Data Imputation: The handling of missing data was stratified based on the proportion of missing values: Variables with > 20% missing data were excluded from subsequent analysis; Multiple imputation was employed for variables with 10–20% missingness; For variables with < 10% missing values, imputation was performed using the mode for categorical variables and the median for continuous ones.

### 2.3 HRCT image acquisition, region of interest segmentation

All image segmentation procedures were performed using the 3D Slicer software platform (version 5.6.1).^1^ The preprocessing pipeline included the following steps: 1. All CT images were resampled to a uniform slice thickness of 1 mm using the “Resample Scalar Volume” module, with intensity values normalized to the range [−1, 1]; 2. Z-score normalization was implemented for image gray values to minimize variability in radiomic features caused by differences in imaging parameters; 3. Automatic segmentation of the bilateral lungs was performed using a threshold-based region growing algorithm, which included vascular and tracheal structures within the lung lobes (window width: 1,250; window level: −875). A total of 13 seed points were strategically placed across multiple anatomical planes: three in the peripheral regions of each lung on the axial plane, three similarly on the coronal plane, and one at the main bronchus.

The initial automated segmentations were manually refined by a radiologist with 5 years of experience in thoracic imaging and subsequently reviewed by a second radiologist with 10 years of expertise. To evaluate segmentation consistency, 20 cases were randomly selected. Interobserver reliability was assessed by comparing segmentations from Radiologist 1 and Radiologist 2. Intraobserver reproducibility was evaluated by having Radiologist 1 repeat the segmentations after a one-month interval. Segmentation consistency was considered acceptable for both inter- and intraobserver comparisons when the intraclass correlation coefficient (ICC) > 0.75.

### 2.4 Radiomics feature extraction, selection and radiomics model develop

**Figure 2:**
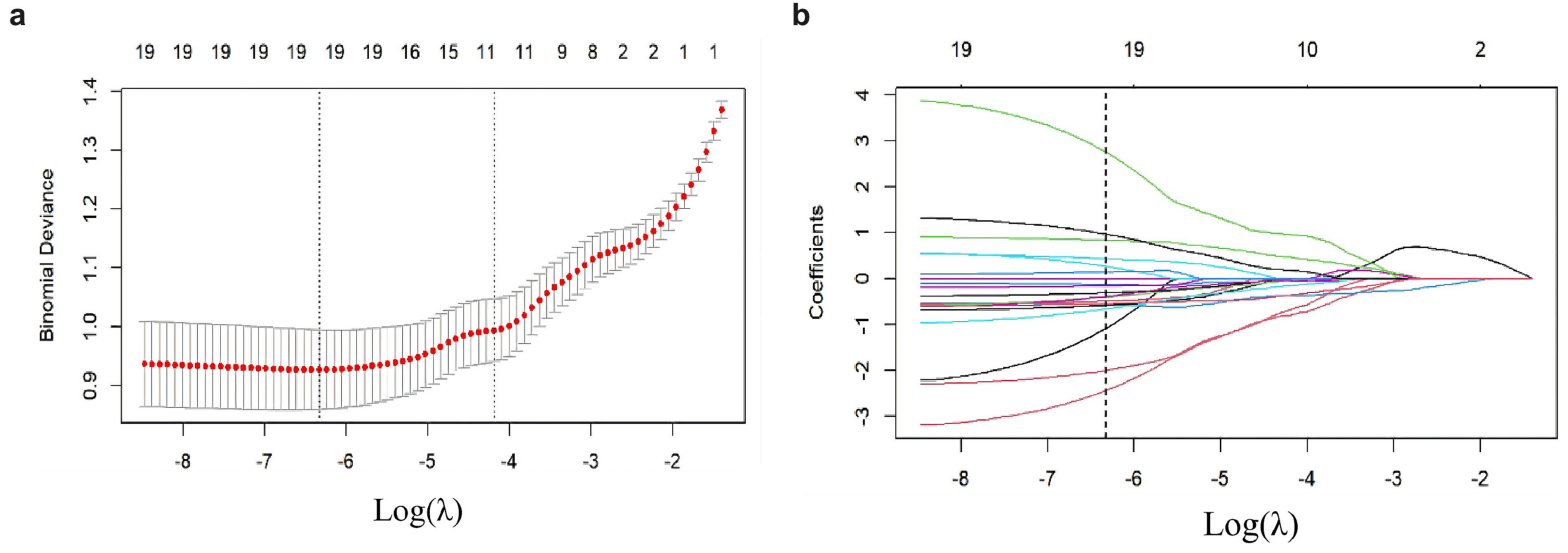
Radiomics feature selection using logistic regression with the LASSO regularization. **(a)** Parameter selection of the LASSO model for 10 cross-validation under the minimum criterion (*λ*). The optimal value of the LASSO tuning parameter (λ) is represented by a vertical line. Draw a dotted line at the optimal value using the minimum standard error of 1. The optimal λ value is 0.002; **(b)** LASSO coefficient distributions for 851 radiomic features. Vertical lines are drawn with 20 selected radiomics characteristic. LASSO: least absolute shrinkage and selection operator.

Feature extraction was performed using the radiomics extension module on 3D slicer software (21). We used minimum redundancy maximum relevance (mRMR) and least absolute shrinkage and selection operator (LASSO) to select the features. Firstly, mRMR was performed to remove redundant and irrelevant features and retain 20 features. Then LASSO was carried out and 20 optimized feature subsets were selected to build the final model (Figure 2). The selected features are then weighted by their coefficients to calculate the rad score.

### 2.5 Development of the clinical factors model

The clinical model was constructed using four clinical parameters, including rash, dyspnea, absolute neutrophil count (ANC) and Alveolar-arterial oxygen gradient (A-a gradient). Univariate analysis was used to compare the differences of clinical factors between the two cohorts, and multiple logistic regression analysis was used to establish a clinical factor model with significant variables in univariate analysis as inputs. The odds ratio for each risk factor was a relative risk estimate with a CI of 95%.

### 2.6 Development of the clinical-radiomics model

In addition, based on multivariable logistic regression analysis, we constructed a clinical-radiomic nomogram model combining radiomic and independent clinical predictors to visually demonstrate the probability of rapid progression of IIM-ILD and evaluate the performance of the combined model. Therefore, we constructed a total of three models to predict the diagnosis of rapid progression of IIM-ILD: a clinical model, a radiomic model, and a combined clinical-radiomic model.

### 2.7 Model validation and comparison

Receiver operating characteristic (ROC) analysis and area under ROC curve (AUC) were used to evaluate the performance of the three models in distinguishing poor prognosis IIM-ILD from non-poor prognosis IIM-ILD in the training and validation groups. Hosmer Lemeshow test was used to measure the goodness of fit of nomogram, and the prediction probability and actual probability of IIM-ILD with poor prognosis were evaluated by correcting curves. By calculating the net benefit for patients under each threshold probability, a Decision curve analysis (DCA) was performed to independently evaluate the clinical value of the model. The accuracy, sensitivity, specificity, positive predictive value and negative predictive value were calculated, respectively.

### 2.8 Statistics

SPSS 23.0 and R version 3.6.3 were used for statistical analysis. The differences between the different groups (finding whether RP-ILD and death endpoint events occurred in the cohort) were calculated using the Mann–Whitney U test and v2 test or Fisher exact test in the continuous and classified data, respectively. The LASSO regression model was analyzed using the “glmnet” software package. ROC curve drawing uses the “pROC” package. Nomogram building using the “rms” package. The Delong test was used to analyze the differences in AUC values between these models. DCA is performed using the ‘rmda’ package. *p* < 0.05 was considered statistically significant.

Given the exploratory nature of this study and the relatively high-dimensional radiomic features (851 initial features, 20 selected after LASSO), formal sample size calculation was not performed. To minimize overfitting, we used LASSO regression with 10-fold cross-validation. The training cohort contained 35 poor-prognosis events, yielding an events-per-variable ratio of 7:1 for the final five predictors (clinical variables plus Rad-score), which is acceptable for exploratory analyses. The smaller validation cohort and the need for external validation in larger, multi-ethnic populations are acknowledged as limitations.

## 3 Results

### 3.1 Clinical features

A total of 167 patients met the diagnostic criteria for IIM-ILD. All patients were divided into a training cohort (*n* = 118) and a validation cohort (*n* = 49) using computer-generated random numbers (Supplementary Table S1). In the training cohort, the prevalence of poor prognosis was 33.1% (39/118); in the validation cohort, it was 32.7% (16/49), with no statistically significant difference (*p* = 0.96). No significant differences in clinical factors were found between the two cohorts (*p* > 0.05). Univariate logistic regression was performed for each candidate variable. Variables with *p* < 0.10 were entered into a multivariate stepwise backward selection model. Multiple logistic regression analysis showed that rash, dyspnea, ANC, and A-a gradient were independent predictors for the clinical model (Table 1). Compared with the good prognosis group, the poor prognosis group was more likely to present with dyspnea and rash, and had higher ANC and A-a gradient levels [rash: odds ratio (OR), 4.17; 95% CI: 1.48–11.79; dyspnea: OR, 3.14; 95% CI: 1.14–8.70; ANC: OR, 1.02; 95% CI: 1.01–1.03; A-a gradient: OR, 1.19; 95% CI: 1.05–1.34] (Table 1). The prevalence of poor prognosis was 33.1% (39/118) in the training cohort and 32.7% (16/49) in the validation cohort, with no statistically significant difference between cohorts (*p* = 0.96; Supplementary Table S1). Univariate analysis of myositis-associated and myositis-specific antibodies (Supplementary Table S2) revealed no statistically significant association with poor prognosis (all *p* > 0.05), although anti-PL-12 (*p* = 0.059) and anti-PL-7 (*p* = 0.081) showed marginal trends. These serological markers were therefore not included in the multivariable model.

**Table 1:** Univariate and multivariate analyses of clinical characteristics associated with poor prognosis in patients with IIM-ILD.

Characteristics	Univariate analysis	Multivariate analysis
	*p*-value OR (95%CI)	*p*-value OR (95%CI)
Rash	0.042* 2.357 (1.031, 5.389)	0.007* 4.17 (1.48, 11.79)
Dyspnea	0.004** 3.305 (1.463, 7.466)	0.027* 3.14 (1.14, 8.7)
ANC	<0.001** 1.196 (1.077, 1.329)	0.006* 1.02 (1.01, 1.03)
A-a gradient	0.001** 1.020 (1.008, 1.032)	0.007* 1.19 (1.05, 1.34)

*p < 0.05, **p < 0.01. *p*-value in multivariate analysis were derived from logistic regression. ANC, absolute neutrophil count; A-a gradient, Alveolar-arterial oxygen gradient.

### 3.2 Feature extraction

A total of 851 radiomics features were extracted from the baseline HRCT images. Following dimensionality reduction using mRMR and LASSO algorithms, 20 optimal features were selected to construct the Rad-score. At the cohort level, there was no significant difference in the Rad-score between the training and validation cohorts (median: −1.1 vs. −1.0, *p* = 0.683), confirming the baseline consistency of the datasets (Supplementary Table S1). However, when stratified by prognosis within each cohort, the Rad-score demonstrated significant discriminative power. In the training cohort, the poor prognosis group exhibited a significantly higher Rad-score compared to the good prognosis group. This discriminatory trend was successfully replicated in the validation cohort (*p* < 0.005), indicating that the Rad-score is a robust indicator of adverse outcomes in IIM-ILD.

### 3.3 Performance of the three models

**Figure 3:**
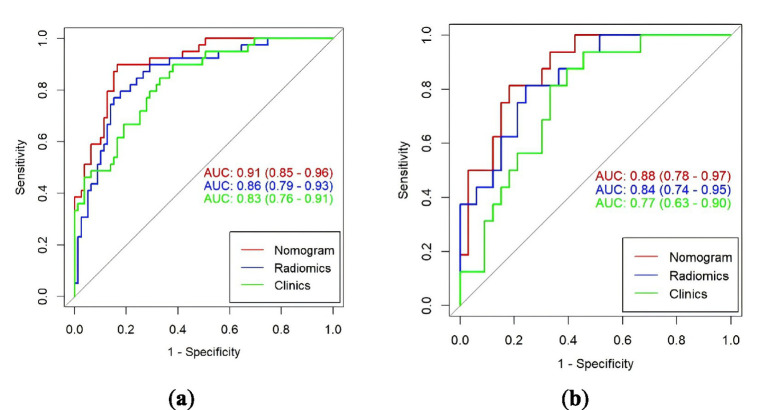
Comparison of ROC curve analyses in three prediction models. **(a)** Training cohort and **(b)** validation cohort. Receiver operating characteristic (ROC) curves of the clinical model (green), radiomics signature (blue), and combined model (red) for predicting poor prognosis in patients with IIM-ILD. AUC, area under the curve; ROC, receiver operating characteristic.

The combined model showed significantly better predictive performance than the clinical model in both the training and validation cohorts. In the training cohort, the AUC was 0.91 (95% CI: 0.85–0.96) for the combined model versus 0.86 (95% CI: 0.79–0.93) for the clinical model (*p* = 0.01). Consistently, in the validation cohort, the combined model achieved an AUC of 0.88 (95% CI: 0.78–0.97) compared to 0.84 (95% CI: 0.74–0.95) for the clinical model (*p* = 0.03; Figure 3).

The radiomics signature showed comparable predictive performance to the clinical model in both cohorts. In the training cohort, the AUC was 0.86 (95% CI: 0.79–0.93) for the radiomics signature versus 0.83 (95% CI: 0.76–0.91) for the clinical model (*p* = 0.58). In the validation cohort, the AUC was 0.84 (95% CI: 0.74–0.95) versus 0.77 (95% CI: 0.63–0.90), respectively (*p* = 0.34; Figure 3).

We further compared the performance of the radiomics signature with that of the combined model. In the training cohort, the combined model achieved an AUC of 0.91 (95% CI: 0.85–0.96), compared to 0.86 (95% CI: 0.79–0.93) for the radiomics signature, although the difference did not reach statistical significance (*p* = 0.06). This comparable performance was confirmed in the validation cohort (combined model AUC: 0.88 [95% CI: 0.78–0.97] vs. radiomics signature AUC: 0.84 [95% CI: 0.74–0.95]; *p* = 0.49; Figure 3).

### 3.4 Construction of the combined model

**Figure 4:**
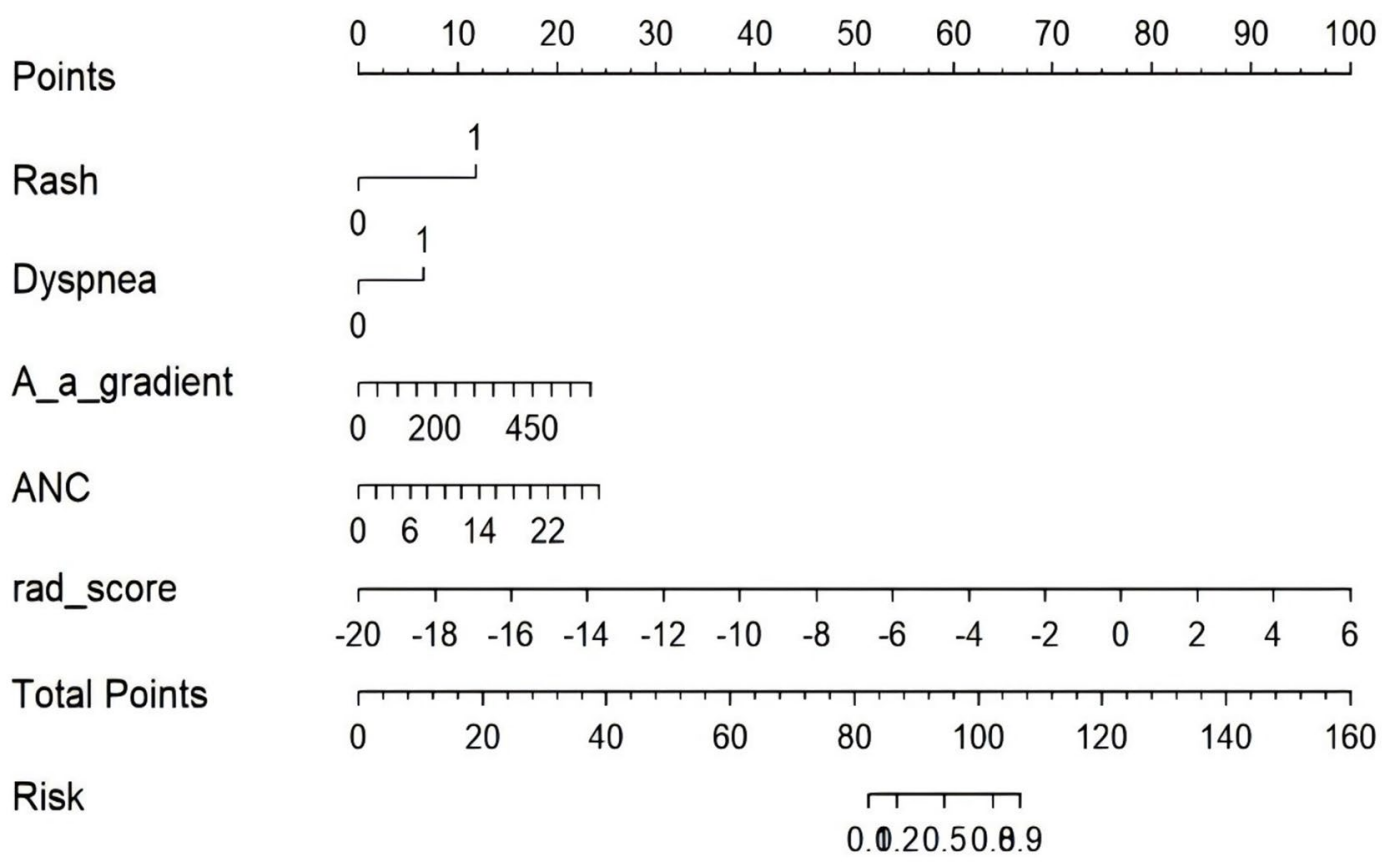
Combined model for predicting the prognosis of patients with IIM-ILD. The combined model integrates five predictors: rash (present vs. absent), dyspnea (present vs. absent), A-a gradient (mL/min), absolute neutrophil count (ANC, ×10^9^/L), and the radiomics signature (rad_score). To use the combined model, locate the patient’s value on each variable axis. Draw a vertical line upward to the “Points” scale to obtain the points for each variable. Sum the points from all five variables and locate the total on the “Total Points” axis. Finally, draw a vertical line downward from the “Total Points” axis to the “Risk” axis to estimate the individual probability of poor prognosis.

**Figure 5:**
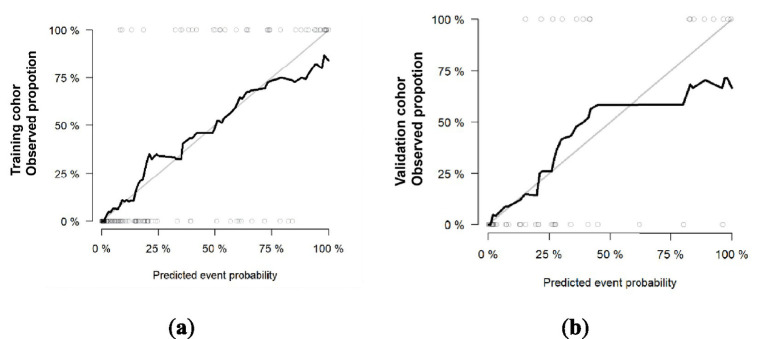
Calibration curves of the combined model for predicting prognosis in IIM-ILD in the **(a)** training and **(b)** validation cohorts. The diagonal gray line represents the ideal perfect prediction, and the black line indicates the combined model performance. Vertical bars represent 95% confidence intervals. The calibration was assessed using the Hosmer–Lemeshow test (training cohort: *p* = 0.59; validation cohort: *p* = 0.57).

**Figure 6:**
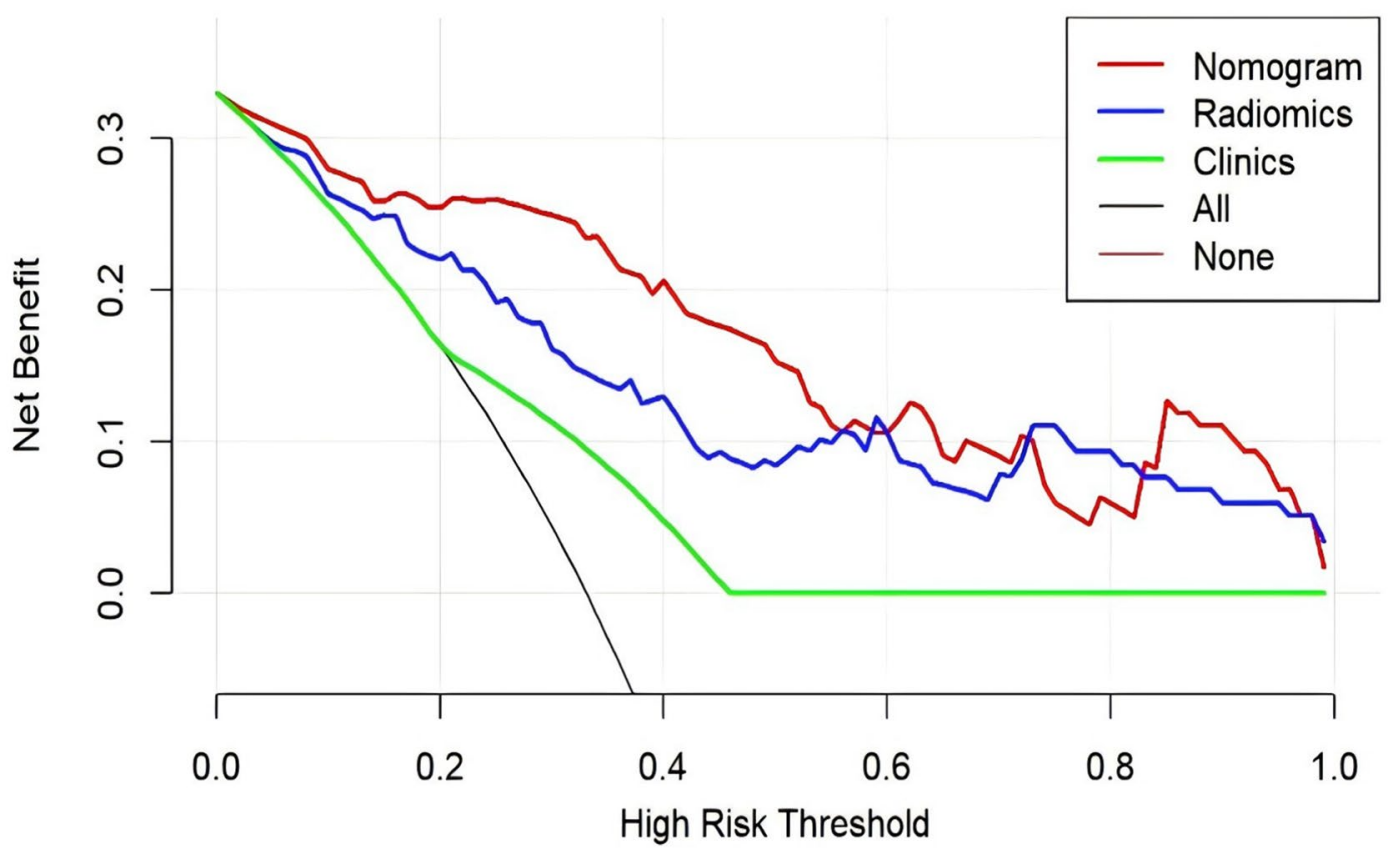
Decision curve analysis for the three predictive models in the training and validation cohorts. The combined model showed the highest net benefit across most threshold probabilities.

The rash, dyspnea, ANC, A-a gradient, and Rad-score were incorporated into the combined model (Figure 4). The predictive performance of the three models is presented in Table 2. The combined model demonstrated superior predictive performance compared to the clinical model with a significantly higher AUC of 0.91 vs. 0.86 (*p* = 0.01) in both cohorts, with higher accuracy, sensitivity, and negative predictive value (Table 2). Calibration curves showed good agreement between predicted and observed probabilities (Figure 5), and the Hosmer–Lemeshow test yielded no significant statistics in either the training (*p* = 0.59) or validation cohort (*p* = 0.57). Decision curve analysis (DCA) demonstrated that the combined model provided greater net clinical benefit than either the clinical model or the radiomics signature alone across a wide range of threshold probabilities (Figure 6).

**Table 2:** Predictive performance of the clinical model, radiomics signature, and combined model.

	ACC(95% CI)	Sensitivity	Specificity	PPV	NPV
Traning cohort						
Clinical model	0.71(0.62–0.79)	0.90	0.62	0.54	0.92
Radiomics model	0.81(0.73–0.88)	0.79	0.82	0.69	0.89
Combined model	0.86(0.78–0.91)	0.90	0.84	0.73	0.94
Validation cohort						
Clinical model	0.61(0.46–0.75)	0.94	0.45	0.45	0.94
Radiomics model	0.73(0.59–0.85)	0.63	0.79	0.59	0.81
Combined model	0.82(0.68–0.91)	0.68	0.90	0.81	0.82

ACC, accuracy; NPV, negative predictive value; PPV, positive predictive value.

## 4 Discussion

The diagnosis and management of idiopathic inflammatory myopathy-associated interstitial lung disease (IIM-ILD) remain challenging due to its heterogeneous clinical presentation and variable prognosis. In this study, we developed and validated a combined model integrating clinical risk factors and radiomic features to predict poor prognosis (AE-ILD or mortality) in patients with IIM-ILD. Our combined model achieved an AUC of 0.91 in the training cohort and 0.88 in the validation cohort, ssignificantly outperforming standalone clinical models. This demonstrates that integrating radiomic features with clinical indicators provides a more comprehensive tool for identifying the ‘high-risk’ phenotype (those prone to AE-ILD or death) compared to traditional methods (22), our radiomics-based approach offers a more interpretable and clinically accessible digital phenotype by decoding sub-visual lung parenchyma heterogeneity.

Our multivariate analysis identified rash (OR 4.17, *p* = 0.007), dyspnea (OR 3.14, *p* = 0.027), ANC (OR 1.02, *p* = 0.006), and A-agradient (OR 1.19, *p* = 0.007) as independent prognostic factors. These findings highlight the critical role of clinical phenotypes in early risk stratification. Cutaneous manifestations, such as Gottron’s signs or heliotrope rash, are not merely diagnostic markers of IIM but are indicators of systemic inflammatory activity and specific immune endotypes (23, 24). Our results align with the CROSS model, which identifies cutaneous involvement as a key predictor for rapidly progressive interstitial lung disease (RP-ILD) in anti-MDA5 + dermatomyositis (23). Furthermore, recent research into antisynthetase syndrome (ASS) endotypes suggests that the severity of skin involvement often correlates with the intensity of systemic cytokine storms, reflecting underlying endothelial damage and severe pulmonary involvement (24). Therefore, the early presence of typical rashes should be viewed as a “red flag” for fulminant pulmonary progression.

Dyspnea represents the subjective manifestation of ventilatory dysfunction and declining gas exchange efficiency. In our model, dyspnea, alongside the A-a gradient (OR 1.19), constitutes a vital dimension for assessing a patient’s physiological reserve. This is consistent with the GAP-ILD model, which posits that physiological variables are indispensable in predicting the survival of ILD patients (25). In IIM-ILD, the exacerbation of dyspnea often precedes significant radiological changes, serving as a clinical surrogate for impaired diffusion capacity and restrictive ventilatory defects.

In our study, Absolute Neutrophil Count (ANC) was identified as a robust independent predictor of poor prognosis. Although patients with clinically documented active bacterial infections were excluded at baseline, approximately 30% of the cohort presented with fever, and elevated ANC remained significantly associated with adverse outcomes. This suggests that the rise in ANC in these IIM-ILD patients may not merely reflect intercurrent infection, but rather indicate intense systemic immune-inflammation. Recent studies have highlighted the role of neutrophil extracellular traps (NETs) in mediating microvascular injury and promoting lung fibrosis in anti-MDA5-positive dermatomyositis (26). Therefore, the prognostic value of ANC in our model likely captures the severity of this neutrophil-mediated sterile inflammation, which contributes to the rapid progression of the disease. The association between elevated ANC and poor prognosis, although modest (OR 1.02), remained statistically significant after adjustment for other factors. However, this finding should be interpreted with caution. First, subclinical infection cannot be fully excluded. Second, high-dose glucocorticoid therapy—which is standard for severe IIM-ILD—induces neutrophilia through demargination, potentially confounding the relationship between ANC and disease activity. Third, while recent studies have implicated neutrophil extracellular traps (NETs) in IIM pathogenesis (27, 28), we did not directly measure NETs or other neutrophil activation markers. Therefore, the observed ANC elevation likely reflects a combination of inflammation, treatment effect, and possibly infection, and should not be overinterpreted as a specific biological driver of poor prognosis.

While traditional HRCT evaluation is often hindered by inter-observer variability and subjective experience, our Rad-score constructed from 20 optimized features provides a standardized, digital phenotype of the disease. Compared to models relying solely on serum biomarkers such as the CROSS model (which focuses on ferritin and anti-MDA5 status) (23), our approach integrates spatial imaging data to more comprehensively reflect the extent of pulmonary involvement. Furthermore, while previous radiomics studies have explored systemic sclerosis (SSc-ILD) (29) or rheumatoid arthritis (RA-ILD) (30), our study specifically addresses the high-risk IIM-ILD subpopulation, filling a critical gap in personalized prognosis.

The clinical utility of our combined model was further confirmed by Decision Curve Analysis (DCA), which demonstrated a high net benefit across a wide range of threshold probabilities. In clinical practice, this allows for the early identification of “high-risk” phenotypes who may require more aggressive interventions. Given that acute exacerbations in IIM-ILD often carry a dismal prognosis, early risk stratification is paramount to initiate intensive therapies, such as high-dose glucocorticoids, immunosuppressants, or early antifibrotic agents.

Despite these findings, several limitations of our study should be acknowledged. First, this was a retrospective study conducted at a single center with a relatively small sample size. The validation cohort in particular is small relative to the dimensionality of radiomic features, raising the risk of overfitting. While LASSO with cross-validation was used to mitigate this, the observed decrease in sensitivity (0.90 to 0.68) and the paradoxical PPV increase suggest that model performance may not generalize reliably. Therefore, our findings should be considered hypothesis-generating, and external validation in large, multi-center cohorts is mandatory before clinical translation. Although domestic multi-center validation is currently ongoing at two other tertiary rheumatology centers in China, external validation in independent, preferably multi-ethnic, cohorts remains essential. We are actively pursuing such collaborations. Second, although myositis-specific antibodies (including anti-MDA5, anti-PL-7, and anti-PL-12) were evaluated in univariate analysis (Supplementary Table S2), none showed a statistically significant association with poor prognosis in our cohort. However, we acknowledge that the sample size may have been insufficient to detect true effects, particularly for less common antibodies such as anti-MDA5. Third, our study only included baseline HRCT scans; the dynamic changes in radiomic features over time remain to be explored. Fourth, the potential biological mechanisms underlying the selected radiomic features were not fully elucidated. Fifth, the manual segmentation of regions of interest is inherently time-consuming and subject to inter-observer variability.

In addition, a fundamental limitation of our radiomics-based approach is its “black box” nature: the 20 selected features lack direct biological interpretability. In a disease with high mortality such as IIM-ILD, clinicians rightly demand transparency when AI informs critical decisions. To address this, future iterations of the model should incorporate explainable AI techniques, including SHAP analysis to quantify feature contributions, and visualization methods such as saliency maps or Grad-CAM to link model outputs to anatomical regions. Additionally, correlating radiomic features with histopathological findings (e.g., organizing pneumonia vs. diffuse alveolar damage) and serum biomarkers (e.g., ferritin, KL-6, MSAs) would enhance biological plausibility. Until such interpretability is achieved, our model should be viewed as a research tool for risk stratification, not a clinical decision-making algorithm. To address these limitations, future research should prioritize the development and implementation of reliable automated segmentation techniques. Such advancements would not only minimize subjective bias and enhance the reproducibility of results but also significantly improve the clinical feasibility and overall robustness of the radiomics workflow, facilitating its integration into routine clinical practice.

## 5 Conclusion

We successfully developed and validated a clinical-radiomics nomogram that provides a robust and non-invasive tool for predicting the prognosis of patients with IIM-ILD. By integrating high-resolution CT radiomic features with key clinical phenotypes—namely rash, dyspnea, ANC, and A-a gradient—this hybrid model significantly outperforms standalone clinical assessments. Our findings offer a novel framework for precise risk stratification, potentially enabling clinicians to identify high-risk individuals early and implement personalized, aggressive treatment strategies. While further multi-center validation and the integration of automated segmentation tools are warranted to enhance workflow robustness, this study underscores the transformative potential of multi-modal data fusion in managing idiopathic inflammatory myopathy.

## Data Availability

The original contributions presented in the study are included in the article/Supplementary material, further inquiries can be directed to the corresponding author.

## Footnotes

1
www.slicer.org

## Glossary

3D: Three-dimensional
A-a gradient: Alveolar-arterial oxygen gradient
AE-ILD: Acute exacerbation of interstitial lung disease
ALC: Absolute Lymphocyte Count
ANC: Absolute Neutrophil Count
ANA: Antinuclear antibody
AUC: Area under the ROC curve
CK: Creatine Kinase
CK-MB: Creatinine kinase MB
CRP: C-reactive protein
DCA: Decision curve analysis
DLCO-SB: Diffusing Capacity of the Lungs for Carbon Monoxide by the Single-Breath Method
DLCO-VA: Diffusing Capacity of the Lungs for Carbon Monoxide per Unit Alveolar Volume
ESR: Erythrocyte sedimentation rate
HRCT: High-resolution computed tomography
ICC: Intraclass correlation coefficient
IIM: Idiopathic inflammatory myopathy
ILD: Interstitial lung disease
LASSO: Least absolute shrinkage and selection operator
LDH: Lactate dehydrogenase
MDA5: Anti-melanoma differentiation-associated gene 5
ML: Machine learning
mRMR: Maximum Relevance Minimum Redundancy
NPV: Negative predictive value
PPV: Positive predictive value
ROC: Receiver operating characteristic
ROI: Region of interest
SSA: Sjogren Syndrome Antigen A
SSB: Sjogren Syndrome Antigen B
TIF1γ: Anti-transcriptional intermediary factor 1-gamma
