# Supplementary material for: Machine learning for predicting acute exacerbation and mortality in idiopathic inflammatory myopathy-associated interstitial lung disease
Source: Front Med (Lausanne). 2026 Jun 10;13:1819663. doi: 10.3389/fmed.2026.1819663 (PMC13290600; doi:10.3389/fmed.2026.1819663)
Supplement: Supplementary file 1 [file Table_1.DOCX]

Appendix A

**Table S1. Baseline comparison of patients with training cohort and internal validation cohort**.

| **Characteristics** | **Training cohort**  **(n=118)** | **Validation Cohort**  **(n=49)** | ***P value*** |
| --- | --- | --- | --- |
| Age, years (Mean ± SD) | 58.5 ± 12.5 | 55.6 ± 10.5 | 0.141 |
| Male gender, n (%) | 32 (27.1) | 13 (26.5) | 1.000 |
| Duration of illness month (Mean ± SD) | 22.3 ± 50.5 | 27.6 ± 66.3 | 0.579 |
| Fever, n (%) | 35 (29.7) | 17 (34.7) | 0.648 |
| Weak, n (%) | 16 (13.6) | 13 (26.5) | 0.073 |
| Myalgia, n (%) | 13 (11.0) | 10 (20.4) | 0.175 |
| Arthritis, n (%) | 26 (22.0) | 20 (40.8) | 0.022 |
| Rash, n (%) | 34 (28.8) | 17 (34.7) | 0.571 |
| Dyspnea, n (%) | 59 (50.0) | 23 (46.9) | 0.849 |
| Cough, n (%) | 65 (55.1) | 23 (46.9) | 0.430 |
| Raynaud’s phenomenon, n (%) | 7 (5.9) | 5 (10.2) | 0.519 |
| ANA ≥ 1:320, n (%) | 66 (55.9) | 32 (65.3) | 0.343 |
| SSA, n (%) | 31 (26.3) | 18 (36.7) | 0.244 |
| RO52, n (%) | 72 (61) | 36 (73.5) | 0.751 |
| SSB, n (%) | 8 (6.8) | 5(10.2) | 0.663 |
| JO1, n (%) | 23 (19.5) | 8 (16.3) | 0.795 |
| PL7, n (%) | 15 (12.7) | 4 (8.2) | 0.565 |
| MDA5, n (%) | 21 (17.8) | 3 (6.1) | 0.086 |
| PL12, n (%) | 6 (5.1) | 7 (14.3) | 0.088 |
| EJ, n (%) | 13 (11.0) | 4 (8.2) | 0.784 |
| TIF1γ, n (%) | 2 (1.7) | 0 (0.0) | 0.892 |
| OJ, n (%) | 4 (3.4) | 0 (0.0) | 0.454 |
| NXP2, n (%) | 2 (1.7) | 0 (0) | 0.892 |
| SPR, n (%) | 6 (5.1) | 1 (2.0) | 0.639 |
| KU, n (%) | 3 (2.5) | 0 (0) | 0.627 |
| MI2, n (%) | 7 (5.9) | 1(2) | 0.500 |
| PM-SCL, n (%) | 2 (1.7) | 2 (4.1) | 0.717 |
| CK (Mean ± SD) | 430.1± 1,064.6 | 613.8 ± 1260.2 | 0.337 |
| CK-MB (Mean ± SD) | 26.3 ± 49.7 | 31.9 ± 69.8 | 0.561 |
| LDH (Mean ± SD) | 377.7 ± 229.8 | 431.1 ± 338.9 | 0.238 |
| ESR (Mean ± SD) | 22.5 ± 16 | 25.4 ± 16.4 | 0.292 |
| CRP (Mean ± SD) | 25.2 ± 46.2 | 16.8 ± 21.9 | 0.227 |
| Ferritin (Mean ± SD) | 539.1 ± 819.3 | 521.6 ± 974.7 | 0.905 |
| ALC (Mean ± SD) | 1.5 ± 0.9 | 1.5 ± 0.8 | 0.544 |
| ANC (Mean ± SD) | 6.1 ± 4.5 | 7.2 ± 3.9 | 0.164 |
| A-a gradient (Mean ± SD) | 54.5 ± 82.2 | 58.5 ± 80.1 | 0.775 |
| BE (Mean ± SD) | 1.6 ± 9.4 | 0.1 ± 1.8 | 0.281 |
| DLCO-SB (Mean ± SD) | 54.3 ± 27.8 | 50.8 ± 12.3 | 0.387 |
| DLCO-VA (Mean ± SD) | 76.5 ± 16.5 | 74.7 ± 14 | 0.511 |
| Radscore (IQR) | -1.1 (-2.3, 0.9) | -1 (-2.6, 0.4) | 0.683 |

Abbreviations: ANA: Antinuclear antibody; SSA: Sjogren Syndrome Antigen A; SSB: Sjogren Syndrome Antigen B; PL7: Anti-threonyl-tRNA synthetase; MDA 5: Anti-melanoma differentiation-associated gene 5; PL12: Anti-alanyl-tRNA synthetase; OJ: Anti-isoleucyl-tRNA synthetase; NXP2: Anti-nuclear matrix protein 2; SRP: Anti-signal recognition particle; TIF1γ:Anti-transcriptional intermediary factor 1-gamma; CK: Creatine Kinase; CK-MB: creatinine kinase MB; LDH: lactate dehydrogenase; ESR: erythrocyte sedimentation rate; CRP: C-reactive protein; ALC: Absolute Lymphocyte Count; ANC: Absolute Neutrophil Count; A-a gradient: Alveolar-arterial oxygen gradient; BE: Base Excess; DLCO-SB: Diffusing Capacity of the Lungs for Carbon Monoxide by the Single-Breath Method; DLCO-VA: Diffusing Capacity of the Lungs for Carbon Monoxide per Unit Alveolar Volume
