# Supplementary material for: Machine learning for predicting acute exacerbation and mortality in idiopathic inflammatory myopathy-associated interstitial lung disease
Source: Front Med (Lausanne). 2026 Jun 10;13:1819663. doi: 10.3389/fmed.2026.1819663 (PMC13290600; doi:10.3389/fmed.2026.1819663)
Supplement: Supplementary file 2 [file Table_2.DOCX]

Appendix B

**Table S2. Univariate analysis of myositis-associated and myositis-specific antibodies for predicting poor prognosis in IIM-ILD.**

| **Antibody** | **OR (95% CI)** | ***P value*** |
| --- | --- | --- |
| SSA, n (%) | 0.65 (0.26–1.53) | 0.336 |
| RO52, n (%) | 1.10 (0.49–2.51) | 0.821 |
| SSB, n (%) | 0.66 (0.09–3.02) | 0.618 |
| JO1, n (%) | 0.68 (0.21–1.95) | 0.497 |
| PL7, n (%) | 2.65 (0.88–8.20) | 0.081 |
| MDA5, n (%) | 0.30 (0.05–1.19) | 0.130 |
| PL12, n (%) | 3.24 (0.96–11.67) | 0.059 |
| EJ, n (%) | 2.21 (0.65–7.57) | 0.196 |
| TIF1γ, n (%) | 2.05 (0.08–52.85) | 0.615 |
| SPR, n (%) | 0.39 (0.02–2.53) | 0.397 |
| KU, n (%) | 1.01 (0.05–10.89) | 0.992 |
| MI2, n (%) | 1.01 (0.14–5.44) | 0.988 |

Abbreviations: ANA: Antinuclear antibody; SSA: Sjogren Syndrome Antigen A; SSB: Sjogren Syndrome Antigen B; PL7: Anti-threonyl-tRNA synthetase; MDA 5: Anti-melanoma differentiation-associated gene 5; PL12: Anti-alanyl-tRNA synthetase; OJ: Anti-isoleucyl-tRNA synthetase; NXP2: Anti-nuclear matrix protein 2; SRP: Anti-signal recognition particle; TIF1γ:Anti-transcriptional intermediary factor 1-gamma; CK: Creatine Kinase; CK-MB: creatinine kinase MB; LDH: lactate dehydrogenase; ESR: erythrocyte sedimentation rate; CRP: C-reactive protein; ALC: Absolute Lymphocyte Count; ANC: Absolute Neutrophil Count; A-a gradient: Alveolar-arterial oxygen gradient; BE: Base Excess; DLCO-SB: Diffusing Capacity of the Lungs for Carbon Monoxide by the Single-Breath Method; DLCO-VA: Diffusing Capacity of the Lungs for Carbon Monoxide per Unit Alveolar Volume
